# Case report: Radiological features of a case of desmoplastic malignant mesothelioma of peritoneum

**DOI:** 10.3389/fonc.2024.1502105

**Published:** 2024-12-11

**Authors:** Jingchao Wang, Heping Deng

**Affiliations:** Department of Ultrasound, Hebei Medical University Third Hospital, Shijiazhuang, Hebei, China

**Keywords:** desmoplastic malignant mesothelioma of peritoneum, malignant peritoneum mesothelioma, primary peritoneal malignancy, peritoneum lesion, abdominal imaging

## Abstract

Desmoplastic malignant peritoneal mesothelioma (DMPM) is an extremely rare and aggressive subtype of sarcomatoid malignant mesothelioma, originating from the mesothelial lining of body cavities. It is characterized by significant local invasiveness and poor prognosis. The nonspecific symptoms of DMPM often result in delayed diagnosis. This case report presents the multimodality imaging findings of DMPM in a 58-year-old male, including ultrasound, CT, contrast-enhanced CT, magnetic resonance imaging (MRI), and 18-fluorodeoxy-glucose positron emission tomography combined with CT (18F-FDG PET/CT). These findings aim to enhance radiologists’ understanding of the imaging features and differential diagnosis of DMPM. In this case, the tumor was located in the right subdiaphragm and the right anterior and left medial lobes of the liver. Due to the patient’s history of alcoholic cirrhosis—a known risk factor for primary liver tumors—the initial diagnostic focus was on identifying a primary liver tumor with potential peritoneal invasion, overlooking other possible etiologies. However, histological results revealed that the liver lesion was secondary to invasion by DMPM. To the best of our knowledge, cases of DMPM invading the liver are exceedingly rare. This report underscores the importance of considering peritoneal tumors in the differential diagnosis when lesions involve both the peritoneum and adjacent organs, despite their rarity.

## 1 Introduction

Desmoplastic malignant peritoneal mesothelioma (DMPM) is a rare and aggressive subtype of malignant peritoneal mesothelioma. Its imaging characteristics align with those typically seen in malignant peritoneal mesothelioma, such as sheet-like thickening of the peritoneum, with no evidence of a primary malignancy, distant metastases, or lymphadenopathy. Due to its nonspecific symptoms and imaging findings, the diagnosis of DMPM is often delayed. In this case, the definitive diagnosis was established through histological findings. Although the optimal treatment for DMPM remains a subject of debate, early diagnosis combined with standard therapeutic approaches—including systemic chemotherapy, cytoreductive surgery, and hyperthermic intraperitoneal chemotherapy—offers potential benefits for patients.

## 2 Case report

A 58-year-old Chinese male with a history of alcoholic cirrhosis was admitted with a one-year history of progressively worsening pain in the right upper quadrant, exacerbated by alcohol consumption and oily foods. The patient reported chronic alcohol abuse but denied asbestos exposure, a history of malignancies, or other significant medical conditions. Physical examination revealed mild tenderness in the right upper quadrant, and laboratory findings were unremarkable apart from a slightly prolonged prothrombin time (13.50 seconds; normal range: 9.4–12.5 seconds). Results from a complete blood count, liver function tests, tumor marker, and pathogens were normal.

**Figure 1:**
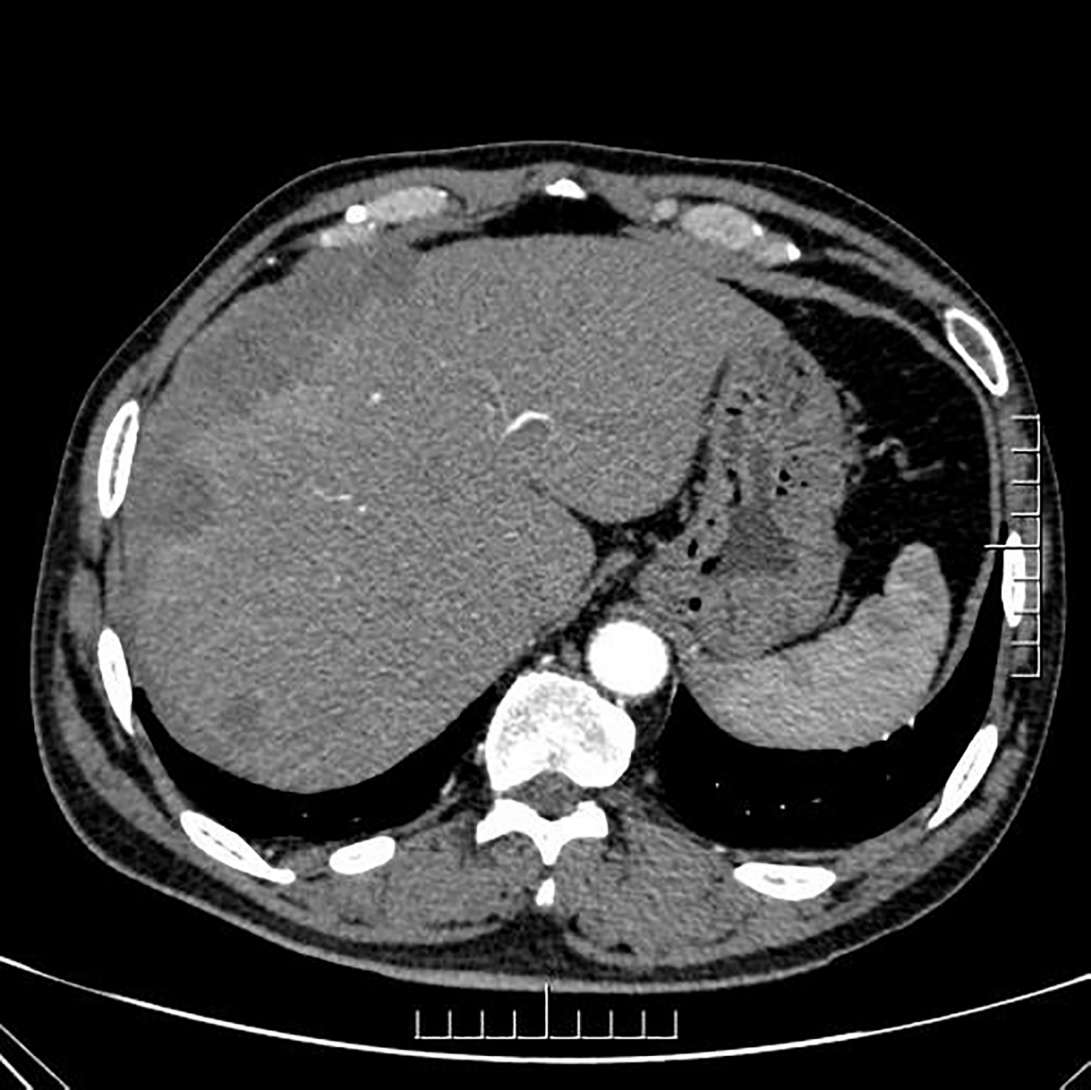
Axial contrast-enhanced CT image of the abdomen during the late arterial phase showing multiple nodular and sheet-like low-attenuation lesions with peripheral enhancement in the right subdiaphragm and right anterior and left medial lobe of the liver.

**Figure 2:**
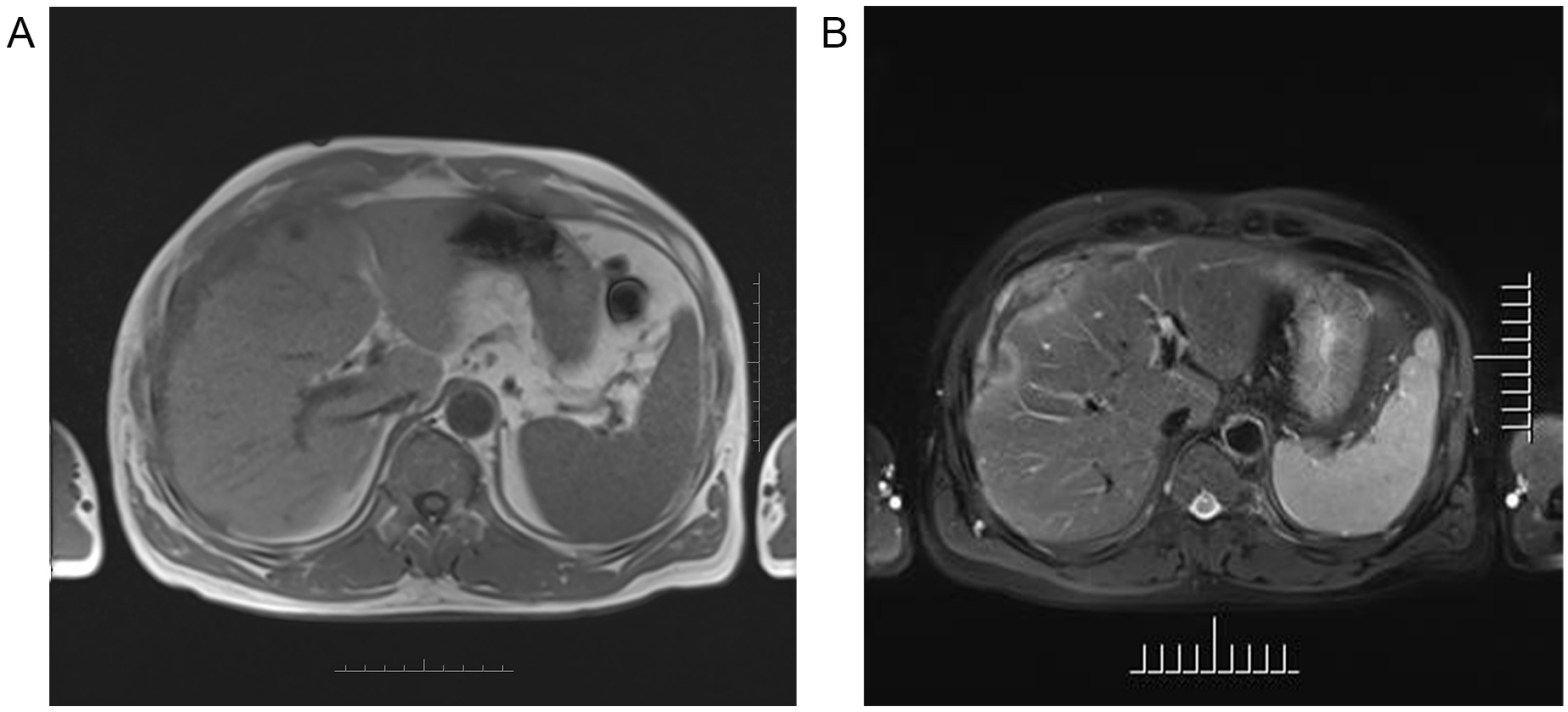
Axial MR images of the upper abdomen showing the lesion in the right subdiaphragm and right anterior and left medial lobe of the liver demonstrated intermediate-to-low intensity on T1-weighted images **(A)**, and central intermediate-to-low intensity and peripherally intermediate-to-high signal on T2-weighted image **(B)**.

**Figure 3:**
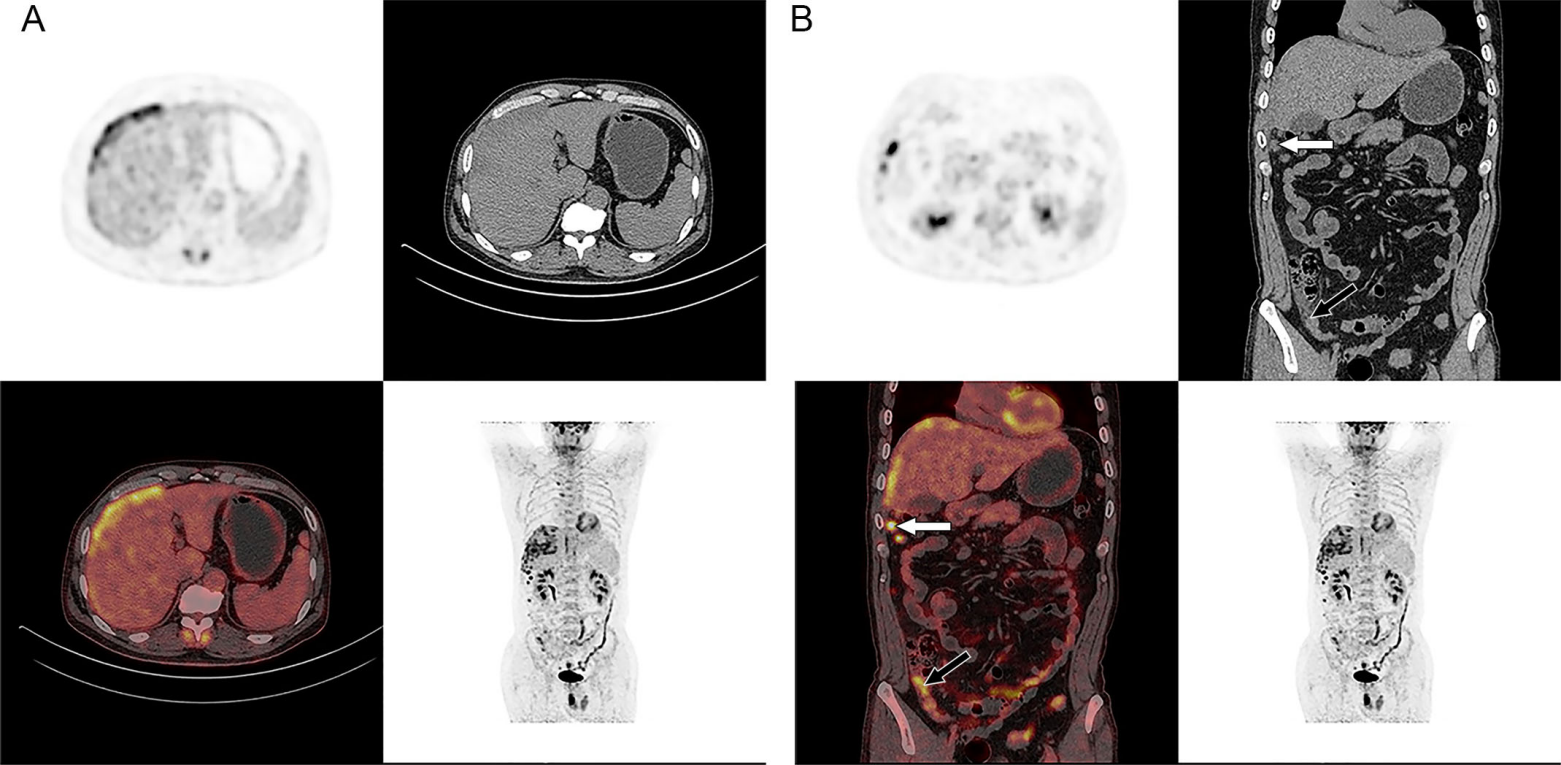
18F-FDG PET/CT images of the whole body. Axial PET/CT images of the abdomen showed abnormally elevated FDG activity in the multiple ill-defined low-attenuation lesions of the right subdiaphragm and right anterior and left medial lobe of the liver (SUVmax of 11.2 and SUVmax of 12.6 in late phase) **(A)**. Coronal PET/CT images of the whole body showed abnormal uptake in the right peritoneum with irregular nodular thickness (black arrow) (SUVmax of 7.7 and SUVmax of 9.3 in late phase) and multiple high activity nodules in the right abdominal cavity (white arrow) (the largest one is SUVmax of 11.9 and SUVmax of 11.6 in late phase) **(B)**.

**Figure 4:**
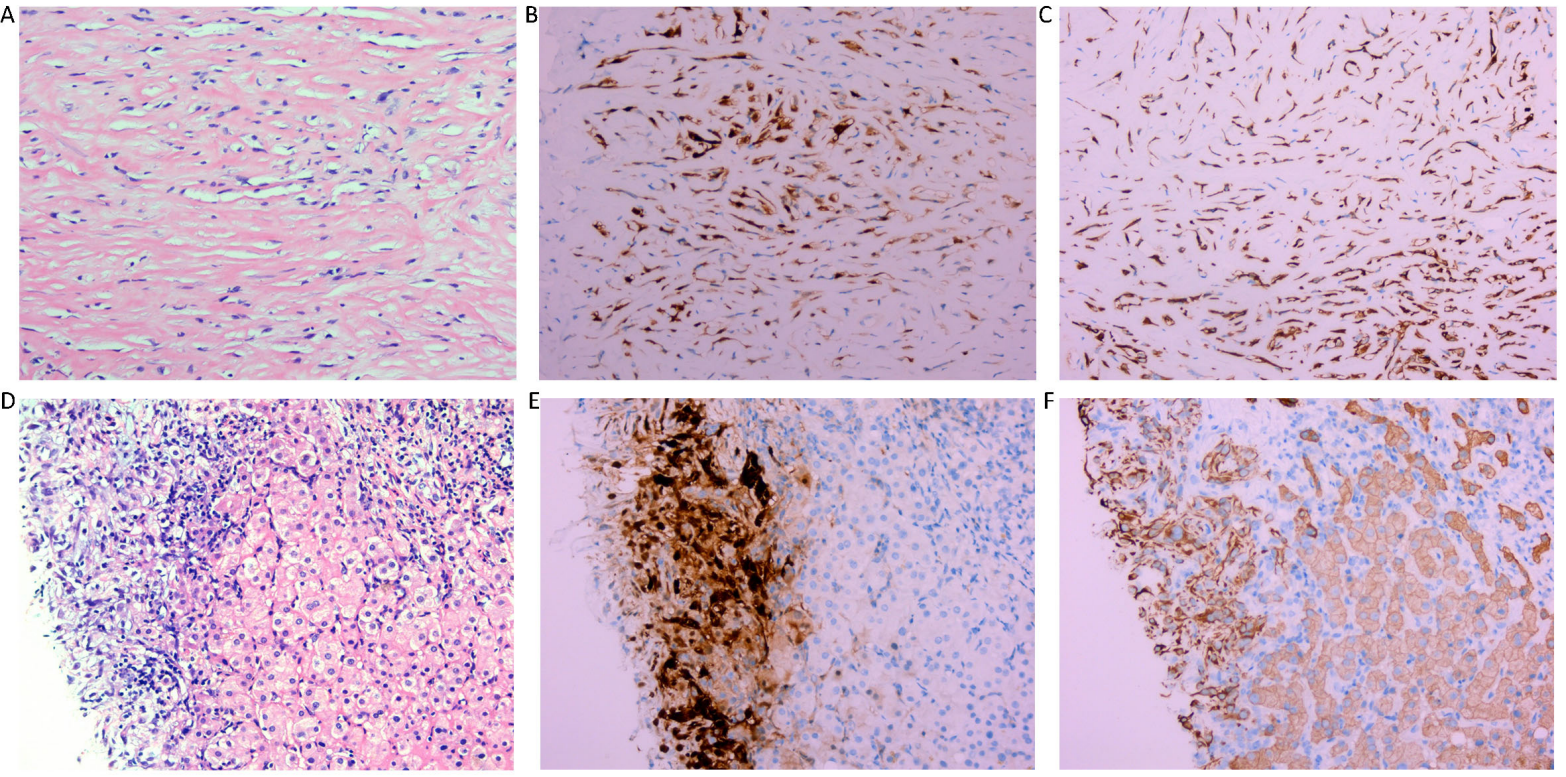
Histological findings of the right subdiaphragmatic lesion. Hematoxylin-eosin (HE) staining (x 200) reveals dense fibrocollagenous tissue infiltrated by spindle-shaped cells **(A)**. and immunohistochemistry revealed immunopositivity for CR and CK **(B, C)**, suggestive of desmoplastic malignant mesothelioma of peritoneum. Histological findings of the lesions of the liver. Hematoxylin-eosin (HE) staining (x 200) reveals dense fibrocollagenous tissue infiltrated by spindle-shaped cells **(D)** and immunohistochemistry showing immunopositivity for CR and CK **(E, F)**, which is indicative of desmoplastic malignant mesothelioma of the peritoneum directedly invading the liver.

Ultrasound (US) imaging revealed an ill-defined, heterogeneous, hypoechoic lesion involving the right subdiaphragm as well as the right anterior and left medial lobes of the liver. The hepatic parenchyma showed diffuse heterogeneity without distinct nodules. Thoracic computed tomography (CT) scans demonstrated an elevated right diaphragm, bilateral pleural thickening, and bilateral diaphragmatic pleural calcifications, along with enlarged anterior mediastinal lymph nodes. No significant pulmonary masses were identified. Abdominal contrast-enhanced CT images in the late arterial phase revealed multiple nodular and sheet-like low-attenuation lesions with peripheral enhancement in the right subdiaphragm, right anterior, and left medial lobes of the liver (**Figure 1**). These lesions showed “washout” in the portal vein and late phases. Enhanced nodules were observed in the peritoneum around the liver and in the fat septa of the ascending colon. Based on thoracic CT findings, the common primary malignancies were excluded, including masses in the lung, pancreas, spleen, kidneys, ureters, and prostate. Magnetic resonance (MR) imaging indicated intermediate-to-low intensity lesions on T1-weighted images (T1WI) and central intermediate-to-low intensity with peripheral intermediate-to-high signal intensity on T2-weighted images (T2WI). Diffusion-weighted imaging revealed restricted diffusion (**Figure 2**). No significant retroperitoneal abnormalities were noted. An 18-fluorodeoxy-glucose positron emission tomography with CT (18F-FDG PET/CT) scan revealed abnormally elevated FDG uptake in the poorly defined low-attenuation lesions in the right subdiaphragm and right anterior and left medial lobes of the liver, with a maximum standardized uptake value (SUVmax) of 11.2. Additional FDG activity was noted in the right peritoneum with irregular nodular thickening (SUVmax 7.7) and multiple hypermetabolic nodules in the right abdominal cavity, the largest of which had an SUVmax of 11.9 (**Figure 3**). No abnormal FDG uptake was observed in the lungs. An ultrasound-guided biopsy of the right subdiaphragmatic lesion and hepatic mass revealed dense fibrocollarous tissue invasion by atypic tumoral cells with spindle, triangular, or polygonal-shaped and hyperchromatic nuclei, involving both the peritoneum and liver (**Figure 4**). Immunohistochemical analysis showed these cells were positive for Ki-67 (20%), cytokeratin (CK) 7, WT-1, vimentin, CK (pan), D2-40 (weakly positive), and calretinin (CR), but negative for villin, CK20, and CDX-2. These findings supported a diagnosis of desmoplastic malignant peritoneal mesothelioma (DMPM). The patient underwent chemotherapy with pemetrexed and cisplatin and was discharged after completing the treatment.

## 3 Discussion

Malignant peritoneal mesothelioma (MPM) is a rare subtype of malignant mesotheliomas originating from mesothelial cells in the peritoneum, accounting for approximately 0.021–0.09% of all malignant tumors (1). While asbestos exposure is the primary risk factor, the association of MPM with asbestos exposure is less clear compared to pleural mesothelioma. Other identified risk factors for MPM include therapeutic irradiation and chronic peritoneal irritation (2). MPM occurs with similar frequency in males and females and is more commonly diagnosed in older individuals, with a median age of 63 years at diagnosis (3).

Based on histological features, MPM is classified into three types: epithelioid, sarcomatoid, and biphasic (4). Desmoplastic malignant peritoneal mesothelioma (DMPM), a rare subtype of sarcomatoid peritoneal mesothelioma, has only been reported in a few cases (5). This subtype is highly aggressive and carries a worse prognosis compared to the epithelioid type (2). DMPM can exhibit local invasion and extend into nearby organs, including the retroperitoneum and peritoneal cavity, or even grow through the diaphragm into the pleural cavity (2). The diagnosis of DMPM is often delayed due to its nonspecific symptoms, with abdominal pain and distension being the most common presentations. In many cases, it is detected incidentally during cross-sectional imaging, abdominal laparoscopy, or laparotomy (6).

Although DMPM is a rare disease, two case reports of DMPM have been published in recent years. Both reports of Badak (7)and Takamaru (8) focus on the disease of DMPM, such as clinical presentation, imaging and laboratory tests, pathological findings, diagnosis, and treatment. Our case is consistent with them, including age of the patients (58, 53, and 74 years), gender (all male), vague symptoms (abdominal distention and abdominal pain), unremarkable medical history and physical examination, except for the patient with ascites reported by Badak. Tumor markers were normal in all patients. Asbestos exposure did not seem to be associated with DMPM. For imaging characteristics, abdominal contrast-enhanced CT and MR imaging of Takamaru’s report showed that the tumor originated from small intestinal mesentery without enhancement and ill-defined borders. In Badak’s report, the abdominal contrast-enhance CT scan and MR imaging demonstrated a 27 × 13× 19 cm fluid collection with enhancing wall structure and internal septation. Two nodules were also found in the liver on CT and MRI. As in our case, the imaging characteristics of the lesion were nonspecific, and histopathologic examination was required to make a definitive diagnosis. In our case, biopsy results indicated dense fibrocollagenous tissue infiltration by spindle-shaped cells involving the peritoneum and liver. These cells were immunopositive for Ki-67 (20%), cytokeratin (CK) 7, WT-1, vimentin, CK (pan), D2-40 (low positive), and calretinin (CR), and were negative for villin, CK20, and CDX-2. The combination of morphology and immunohistochemistry led to the diagnosis of DMPM, which is consistent with the results of Hui’s study (9). Compared with the reports of Badak and Takamaru, our case presents the multimodality imaging findings of DMPM, including ultrasound, CT, contrast-enhanced CT, MR imaging, and 18F-FDG PET/CT, to enhance radiologists’ understanding of the imaging features and differential diagnosis of DMPM.

The imaging characteristics of DMPM are nonspecific and do not allow for accurate prediction of the histological subtype (10). However, these imaging features are consistent with those of MPM, including sheet-like thickening of the peritoneum, absence of primary malignancy or distant metastasis, and lack of lymphadenopathy (11). DMPM can metastasize to the abdominal wall, adjacent organs, retroperitoneum, and through the diaphragm into the pleural cavity (12). Ascites, calcifications, and lymphadenopathy are typically uncommon (2). When numerous calcifications are present within a peritoneal mass, alternative diagnoses should be considered. CT imaging of DMPM typically reveals plaque-like or sheet-like diffuse peritoneal thickening, peritoneal and mesenteric nodules, and omental caking (13). CT is advantageous in differentiating peritoneal mesothelioma from other peritoneal diseases, evaluating lesion resectability, and assessing treatment response. However, the contrast-enhanced CT appearance of peritoneal thickening and nodules is often nonspecific, with the lesions appearing either homogeneous or heterogeneous. MR imaging typically shows intermediate-to-low signal intensity on T1-weighted images (T1WI), intermediate-to-high signal intensity on T2-weighted images (T2WI), and diffusion restriction (11). Lesions often demonstrate high 18F-FDG uptake with diffuse, focal, or mixed distribution patterns (14).

The differential diagnosis for peritoneal diseases includes peritoneal carcinomatosis (PC), peritoneal lymphomas, and infectious peritonitis. PC refers to the implantation of malignant tumors from primary sites, most commonly the colon, ovaries, and stomach. Ascites is frequently associated with advanced PC, and its presence can help identify the primary malignancy (15). Peritoneal masses in PC may appear as solid or cystic. On CT, the peritoneum can exhibit thickening with low-density masses. Mucinous neoplasms may display specks of calcification. Solid masses appear hypointense on T2WI but hyperintense on diffusion-weighted imaging (DWI), while cystic masses demonstrate hyperintensity on T2WI and hypointensity on DWI. Contrast-enhanced CT and MRI are vital for PC assessment, with masses often visible in venous or delayed phases (16). Peritoneal lymphomas, often associated with diffuse large B-cell lymphoma and Burkitt’s lymphoma, present with omental caking and homogeneous bulky masses. CT typically reveals diffuse retroperitoneal lymphadenopathy and mild-to-moderate ascites (17). FDG avidity varies in lymphomas, depending on their metabolic activity, with indolent lymphomas showing low-grade FDG uptake (18). Other primary peritoneal tumors, such as primary peritoneal serous carcinoma and desmoplastic small round cell tumor, can exhibit similar imaging features, including peritoneal omental thickening or nodularity with hypointensity on T1WI and hyperintensity on T2WI. Thus, histological examination is essential for a definitive diagnosis of peritoneal diseases. Infectious or inflammatory peritonitis usually presents with smooth peritoneal thickening, whereas malignant tumors often demonstrate irregular, nodular peritoneal thickening. Granulomatous peritonitis, typically caused by tuberculous peritonitis (accounting for 90% of cases), is characterized by peritoneal granulomatous inflammation. CT imaging of tuberculous peritonitis shows smooth peritoneal thickening and tiny nodules. Contrast-enhanced CT demonstrates significant enhancement of the peritoneum, while DWI indicates restricted diffusion of the nodules (19). Due to its high invasiveness and potential for peripheral metastasis, the prognosis for DMPM remains poor. The optimal therapeutic approach is still controversial, but early diagnosis and a combination of systemic chemotherapy, cytoreductive surgery, and hyperthermic intraperitoneal chemotherapy may improve patient outcomes (20).

In the present case, the diagnosis of DMPM was confirmed through histological analysis. Notably, the mass was located in the right subdiaphragm and the right anterior and left medial lobes of the liver. Initially, the diagnostic focus for this patient with alcoholic cirrhosis—a known risk factor for primary liver tumors—centered on a liver-originating tumor with potential peritoneal invasion. However, due to the absence of supportive evidence on contrast-enhanced CT and negative serum tumor marker findings, a biopsy was performed to establish a definitive diagnosis. Histology revealed the peritoneal lesion as the primary tumor, with liver invasion by DMPM. To the best of our knowledge, reports of DMPM invading the liver are exceedingly rare. This case underscores the importance of considering peritoneal tumors when lesions involve both the peritoneum and adjacent organs, even though such tumors are relatively uncommon. Radiologists must remain vigilant and not overlook this potential diagnosis in their evaluations.

## 4 Conclusion

DMPM is a rare aggressive subtype of malignant peritoneal mesothelioma. This case is a challenge for radiologists. First, its rarity means that many radiologists may be unfamiliar with its imaging characteristics. Second, no single imaging modality provides definitive or characteristic features for diagnosis. A combination of imaging modalities—such as ultrasound, contrast-enhanced CT, MR, and PET-CT—is essential to support the diagnostic process. These modalities collectively help evaluate the extent and severity of lesions, providing critical information for determining appropriate clinical treatment strategies.

## Data Availability

The original contributions presented in the study are included in the article/supplementary material. Further inquiries can be directed to the corresponding author.
